# Wheat straw decomposition constituents: a genome-wide association study and environmental influence analysis

**DOI:** 10.3389/fpls.2026.1837289

**Published:** 2026-07-16

**Authors:** Nathan S. Nielsen, Melinda Zubrod, Tami L. Stubbs, Kimberly Garland-Campbell, Arron H. Carter

**Affiliations:** 1 Pace-Nielson Farms, Parma, ID, United States; 2 Washington State University, Department of Crop and Soil Sciences, Pullman, WA, United States; 3 Washington State Conservation Commission, Olympia, WA, United States; 4 Wheat Genetics, Quality, Physiology, and Disease Research Unit, Agricultural Research Service, United States Department of Agriculture, Pullman, WA, United States

**Keywords:** genome-wide association study, marker-trait association, no-till production, straw decomposition, wheat

## Abstract

Adoption of no-till farming in Eastern Washington has been slow due to difficulties managing wheat (*Triticum aestivum* L.) straw residue in no-till systems. We hypothesize that a genome-wide association study (GWAS) will identify single nucleotide polymorphisms (SNPs) that can assist in understanding the genetic loci associated with straw decomposition. The straw from a panel of 465 soft white winter wheat cultivars in the Pacific Northwest was harvested over two years at three locations throughout Eastern Washington. The samples were analyzed for several decomposition constituents, including neutral detergent fiber (NDF), acid detergent fiber (ADF), acid detergent lignin (ADL), cellulose, and hemicellulose using a wet chemistry procedure whereas C was determined using dry combustion. Significant differences among environments were observed for all fiber and chemical constituents, with environment accounting for the largest source of variation across all traits. Broad-sense heritabilities were correspondingly low (H^2^ = 0.03-0.21), reflecting the strong influence of environmental conditions on straw composition. Genotyping was performed using the 90K Illumina SNP chip and a GWAS was conducted using Fixed and random model Circulating Probability Unification (FarmCPU) implemented in the statistical program R. Twenty-three marker-trait associations were identified across 12 chromosomes. Cellulose, NDF, and ADF were the most useful traits for identifying chromosomal loci of interest. Five significant loci were identified on chromosomes 1B, 2B, 4B, 5B, and 6B. Four of those loci were associated with cellulose, three with NDF, and three with ADF. The distribution of the significant SNPs across chromosomes, however, demonstrates the genetic complexity of these straw breakdown constituents.

## Introduction

Straw decomposition is a crucial factor in no-till crop production systems. No-till is an important practice that conserves soil, reduces erosion, improves water retention, and enhances soil health by minimizing soil disturbance (Ruan and Robertson, 2020). Crop residue plays a critical role in no-till systems by covering and protecting the soil from erosion, maintaining moisture, and supporting beneficial organisms (Nurbekov et al., 2024). Accumulated runoff and soil erosion have been observed to be up to 54 times lower in a no-till system than in a conventional tillage system (Williams et al., 2014). Managing crop residue is crucial as excessive accumulation can prevent seed-to-soil contact, delay soil warming, and prevent seedling emergence; therefore, balanced residue management is essential for effective no-till farming (Kravchenko and Thelen, 2007). Developing wheat cultivars with genetic traits that promote appropriate rates of straw decomposition could help support no-till adoption by improving residue management across different production environments.

The breakdown of straw is highly dependent on environmental conditions, particularly moisture levels. In areas with high rainfall, straw decomposes more rapidly due to increased microbial activity that thrives in moist conditions (Rosario-Lebron et al., 2019). A combination of heavy tillage, clay soils, and steep slopes makes the high rainfall (>300mm), Palouse region of eastern Washington prone to serious water erosion (Frazier et al., 1983) and puts future productive stability at risk (Schillinger and Papendick, 2008). In the low rainfall (<250mm) region of eastern Washington, a winter wheat/fallow rotation is utilized to conserve moisture. Adoption of no-till practices has been slow in eastern Washington due to limited summer rainfall delaying straw decomposition. Tillage is conducted during the fallow period during summer months to control weeds. This tillage breaks the capillary pores in the soil and creates a dust mulch, which is prone to intense wind erosion (Papendick et al., 1973; Sharratt et al., 2007). Furthermore, eastern Washington receives that majority of its annual rainfall in the winter months, which is not conducive to straw breakdown in the summer and fall (Maaz et al., 2017).

Depending on the cropping system each grower is using, the needs for straw residue management will differ. In high rainfall regions in the Eastern U.S., the straw remaining in no-till production systems decomposes rapidly over the winter months avoiding the planting complications that excessive straw residue can create (Aher et al., 2017). On the other hand, straw residue in the low rainfall regions needs to decompose slowly to cover the soil during the entirety of the fallow season. Straw residue breakdown depends largely on plant cell wall composition; therefore, identifying the genetic loci associated with straw decomposition will facilitate the breeding of cultivars with desirable straw characteristics. Different winter wheat cultivars have demonstrated variation in straw breakdown capacity (Stubbs et al., 2009). Plant cell wall composition is complex, and the genetic basis of its synthesis remains incompletely understood (McCann and Rose, 2010).

Several fiber and chemical constituents of wheat have previously been associated with straw decomposition (Stubbs et al., 2009; Douglas et al., 1980). Two structural components of plant cell walls – hemicellulose and cellulose (Wagner and Wolf, 1998) - are quantified through a wet chemistry procedure that measures neutral detergent fiber (NDF), acid detergent fiber (ADF), and acid-detergent lignin (ADL). Carbon (C) is determined through dry combustion analysis. These analytical methods are time-intensive and costly, limiting their application to large-scale cultivar screening for decomposition potential.

Knowledge of the genetic factors contributing to straw breakdown will be facilitated by a genome-wide association study (GWAS). The GWAS has potential to identify molecular markers that are statistically associated with the fiber and chemical constituents used to predict decomposition potential. A GWAS can also contribute to the understanding of specific functional genes that are involved in straw breakdown in wheat. To the best of our knowledge, no MTAs or quantitative trait loci (QTL) have been reported specifically for the fiber and chemical constituents of winter wheat straw, although related work in spring (Joshi et al., 2019) and tetraploid wheat (Esposito et al., 2022) has identified MTAs for ADF, ADL, NDF, and cellulose across multiple chromosomes, with modest per-marker effect sizes and low broad-sense heritabilities consistent with polygenic architecture. Additional QTL for ADF and NDF have also been identified in barley straw (Grando et al., 2005).

Therefore, the objectives of this study were to 1) phenotype a diverse winter wheat panel grown across multiple environments and analyze the environmental effects on the production of NDF, ADF, ADL, cellulose, hemicellulose, N, and C in straw and 2) perform a GWAS to identify molecular markers that have associations with the constituents of straw decomposition.

## Materials and methods

A panel of 465 soft white winter wheat cultivars from the Pacific Northwest (PNW) was assembled to represent the genetic diversity in the region’s breeding programs (Washington State University, Oregon State University, University of Idaho, USDA-ARS, private breeding companies) and commercially available cultivars. This panel has been used for multiple other GWAS studies (Froese and Carter, 2016; Jernigan et al., 2018; Liu et al., 2018). Genotyping and population structure were determined previously as presented in Jernigan et al. (2018). A total of 81,575 SNP markers were obtained using an Illumina Infinium iSelect 90K SNP chip. After those with more than 20% missing data and those with minor allele frequency (MAF) < 0.05 were filtered out, 15,229 high quality markers remained for use. Chromosome position of the SNPs was determined using the consensus map created by Wang, S. et al. (2014). Also included in the genotypic dataset was an SNP associated with the resistance gene, *Pch1*, to eyespot (caused by *Oculimacula yallundae* and *O. acuformis*) which is located on chromosome 7D (Worland et al., 1988). Similarly, a SNP associated with the *Rht-B1* semi-dwarfing gene on chromosome 4B (Pearce et al., 2011) was added. Both loci were included because they have documented effects on straw composition and architecture in wheat. Those genes were assayed using competitive allele specific markers (KASP) (https://maswheat.ucdavis.edu).

### Sample collection and experimental design

This panel was grown and harvested in Pullman, WA in 2016 and Pullman, Central Ferry, and Mansfield, WA in 2017. Pullman is located at 46.73° N, 117.18° W, on the eastern border of Washington state, and the elevation is 717 m above sea level. The average annual precipitation exceeds 500 mm, soil types range from silt loams to silty clay loams and are part of the Palouse soil series (USDA-NRCS, 2017), and winter wheat grain yields average around 7900 kg/ha (Wheat & Small Grains, 2018). Central Ferry is located approximately 95 km southwest of Pullman at 46.62° N, 117.79° W, and averages approximately 6700 kg/ha of grain yield under 600 mm of irrigation. It sits at an elevation of 195 m on silt loam soils classified mostly under the Chard soil series (USDA-NRCS, 2017). Mansfield, located approximately 320 km northwest of Pullman at 47.81° N, 119.64° W, has an annual precipitation of less than 300 mm and grain yields average around 3550 kg/ha (Wheat & Small Grains, 2018). The soils are mostly part of the Touhey soil series, classified as ashy fine sandy loam (USDA-NRCS, 2017), and the elevation is 692 m. An augmented design was used for this population with repeating checks every 20 entries and one replication per location. The repeated checks were not included in the analysis. One half meter row of straw from each cultivar in the panel was harvested at ground level at harvest maturity (stage 11.4 on Feekes’ scale) (Large, 1954; Feekes, 1941) and the heads were removed. For consistency, the leaves and nodes were also removed from the samples. The remaining internode portion was cut into 1–2 cm pieces and then ground to pass a one mm sieve using a FOSS Cyclotec 1093 (FOSS North America, Eden Prairie, MN).

### Fiber and nutrient analysis

Specialized filter bags (ANKOM Technology, Macedon, NY) were used to enclose 0.5-0.55 grams of ground winter wheat straw for NDF, ADF, and ADL analyses using the van Soest et al. (1991) procedure with ANKOM automated systems (ANKOM Technology, Macedon, NY). The NDF procedure removed starches, sugars, free amino acids, and other water-soluble components, leaving hemicellulose, cellulose, and ADL. The ADF procedure removed the hemicelluloses, leaving only the cellulose and ADL. The ADL procedure removed the cellulose from the straw. The NDF and ADF procedures were performed sequentially using an ANKOM 200 Fiber Analyzer (ANKOM Technology, Macedon, NY) and following ANKOM procedures. The straw samples were then digested in 72% H_2_SO_4_ to determine the ADL. After each procedure, the samples were dried overnight in a lab hood and then were dried in a hybridization incubator for a minimum of 6 h at 64 °C. The samples were removed from the incubator, placed in plastic bags with desiccators, and individually weighed. The hemicellulose value was calculated as the difference between NDF and ADF whereas the cellulose value was determined by calculating the difference between ADF and ADL. Dry combustion with a LECO TruSpec Analyzer (LECO Corp., St. Joseph, MI) was used to determine C as described by Gazulla et al. (2012).

### Statistical analysis

Prior to any statistical analysis, influential outliers were removed based upon analysis of the residuals. Summary statistics were calculated in R (R Foundation for Statistical Computing, Vienna, Austria) using a one-way analysis of variance (ANOVA) and Tukey *post hoc* test with the package multcomp (Hothorn et al., 2008). Pearson correlations among traits were calculated in JMP Genomics (SAS Institute Inc., Cary, NC, USA). The R-package GAPIT2 was used for GWAS (Tang et al., 2016). Population structure present in this panel (Jernigan et al., 2018) was accounted for by using principal component analysis (PCA) calculated in GAPIT2. For each trait, the number of principal components used was determined by analyzing the quantile-quantile (Q-Q) plots and the best fit for the model was selected. Principal components one and three were used for NDF, ADF and C. Principal components two and three were the best fit for ADL. The first principal component was used for cellulose. Zero principal components were used for hemicellulose, indicating that the population structure within the sampled cultivars did not have a significant influence on this trait. Multiple environment analyses were conducted using linear models to calculate best linear unbiased predictions (BLUPs) using the lme4 (Bates et al., 2015) package in R with both genotype and environment included as random effects. The marker-trait association analysis was performed using a mixed linear model (MLM) in Fixed and random model Circulating Probability Unification (FarmCPU) (Liu et al., 2016) and implemented in GAPIT2 (Tang et al., 2016). To correct for the use of multiple statistical tests, the Bonferroni correction method was applied with a significance level of α = 0.05. Broad-sense heritability (H^2^) was calculated for each trait as the ratio of total genetic variance to total phenotypic variance (Holland et al., 2003) based on mixed models analysis using the MIXED procedure of SAS 9.3 (SAS Institute Inc., Cary, NC, USA). The four environments were included in the calculation as replications. Genotype + genotype-by-environment interaction (GGE) biplots were developed in R using the GGEBiplotGUI package (Bernal, 2023).

### Cross-study marker alignment

To enable direct positional comparison between SSR-based QTL identified by Verma et al. (2005) and the SNP markers identified in the present study, both marker sets were anchored to a common physical reference. SSR primer sequences for the markers flanking the Verma et al. (2005) lodging QTL on chromosomes 2B, 4B, and 7D were obtained from GrainGenes BLAST porta, with short-sequence parameters (-task blastn- short, word size 7, E E-value threshold 1000) applied to the SSR primers. For each query, the top hit on the expected chromosome was taken as the genomic position. SSR marker positions were defined as the midpoint between the forward and revers primer hits.

## Results

### Trait statistics and correlations

Pullman averaged higher NDF values than the other locations in 2016 and 2017 (**Table 1**). The environment that had the lowest average NDF value was Mansfield 2017. Pullman had the highest average ADF in 2017 and the highest average ADL in 2016, although both years were similar. Mansfield 2017 also had the lowest average ADF and ADL values. Cellulose was highest in Pullman 2017 and lowest in Mansfield 2017. Pullman 2016 had the greatest average hemicellulose and Central Ferry 2017 averaged the lowest. Most traits differed significant among all four environments (**Table 2**; **Supplementary Figure 1**). The only exceptions were ADF, hemicellulose, and carbon. Pullman did not have a significant difference in ADF between 2016 and 2017. There also was no difference in hemicellulose content between Pullman 2016 and Mansfield 2017. No significant difference was observed in carbon between Pullman 2017 and Mansfield 2017. All other pairwise comparisons within a trait were significant at P<0.05, as indicated by differing superscript letters in **Table 1**.

**Table 1:** Summary statistics for fiber and nutrient constituents of straw decomposition across four environments in Eastern Washington.

Pullman 2016						
	NDF	ADF	ADL	CELL	HEMI	C
	————————————— % ——————————————					
Min	73.02	46.83	5.06	40.77	24.28	44.24
Max	88.29	59.57	9.27	51.90	31.35	47.76
Mean	82.86^a^	54.57^a^	7.09^a^	47.48^b^	28.29^a^	46.15^a^
SD	2.25	2.16	0.78	1.73	1.18	0.52
Central Ferry 2017						
	NDF	ADF	ADL	CELL	HEMI	C
	————————————— % ——————————————					
Min	64.41	39.94	4.08	34.59	22.56	39.39
Max	82.73	57.31	9.13	48.74	30.90	45.93
Mean	76.32^c^	49.93^b^	6.24^c^	43.66^c^	26.45^c^	43.28^b^
SD	3.08	2.97	0.86	2.38	1.32	1.21
Pullman 2017						
	NDF	ADF	ADL	CELL	HEMI	C
	————————————— % ——————————————					
Min	76.39	49.59	4.96	43.25	23.76	39.90
Max	86.34	60.14	9.98	51.13	31.33	45.24
Mean	82.40^b^	54.89^a^	6.89^b^	47.99^a^	27.54^b^	41.98^c^
SD	1.61	1.73	0.85	1.37	1.16	1.31
Mansfield 2017						
	NDF	ADF	ADL	CELL	HEMI	C
	————————————— % ——————————————					
Min	63.64	40.43	3.79	35.20	23.72	38.75
Max	83.78	53.46	8.03	46.23	33.26	45.59
Mean	75.22^d^	47.06^c^	5.50^d^	41.56^d^	28.14^a^	41.87^c^
SD	3.16	2.38	0.69	2.00	1.53	1.84
	
*H^2^‡*	0.11	0.15	0.18	0.21	0.15	0.09

† NDF, neutral detergent fiber; ADF, acid detergent fiber; ADL, acid detergent lignin; CELL, cellulose; HEMI, hemicellulose; C, carbon.

‡ Denotes broad-sense heritability for all fiber and chemical traits estimated across all four environments; values are not environment-specific.

Minimum, maximum, mean, standard deviation, and broad-sense heritability are reported for each fiber and nutrient constituent. Means within a column followed by the same letter are not significantly different at P>0.05 (Tukey’s HSD); all other pairwise comparisons within a trait are significant at P<0.05. †.

**Table 2:** Variance components and standard deviation calculated using linear mixed models for the fiber and chemical constituents of straw decomposition.

Trait†	Component‡	Variance§	SD¶
**NDF**	Cultivar	0.7869	0.8871
	Environment	15.9654	3.9957
	Residual	5.956	2.4405
**ADF**	Cultivar	0.8449	0.9192
	Environment	14.375	3.7914
	Residual	4.6806	2.1635
**ADL**	Cultivar	0.1186	0.3444
	Environment	0.519	0.7204
	Residual	0.5234	0.7235
**CELL**	Cultivar	0.7683	0.8765
	Environment	9.5259	3.0864
	Residual	2.8436	1.6863
**HEMI**	Cultivar	0.2559	0.5059
	Environment	0.6963	0.8345
	Residual	1.4425	1.201
**C**	Cultivar	0.1742	0.4174
	Environment	4.0155	2.0039
	Residual	1.5347	1.2388

† NDF, neutral detergent fiber; ADF, acid detergent fiber; ADL, acid detergent lignin; CELL, cellulose; HEMI, hemicellulose; C, carbon.

‡ Components of variation for each trait.

§ Variance for each component. The highest value indicates the source of most variation within traits.

¶ Standard deviation of each variance component.

A similar pattern was observed in trait correlations across all four environments (**Supplementary Table 1**). The strongest correlation across all environments was generally between ADF and cellulose, which ranged from 0.87 – 0.97. Strong correlations were also observed between NDF and cellulose (R^2^ = 0.76 – 0.93), as well as NDF and ADF (R^2^ = 0.73 – 0.91). ADF and ADL had a correlation coefficient of 0.76 in Central Ferry 2017 but were less correlated in the other three environments. Correlations of carbon to all other traits was very low whereas hemicellulose and N were often negatively correlated with the other traits.

The variation for each trait differed across environments, as evidenced by the standard deviations (**Table 1**). Central Ferry 2017 displayed the most variation for several traits, including ADF, ADL, cellulose, and N, whereas the greatest variation in NDF, hemicellulose, and C was observed in Mansfield 2017. Pullman 2017 generally displayed low variation in the traits, as was observed for NDF, ADF, cellulose, and hemicellulose. For all traits, the greatest source of variation came from the environment (**Table 2**). As a consequence of the large environmental variation, broad-sense heritabilities were low for each trait (**Table 1**).

A challenge faced by breeders when selecting for these fiber and chemical components is that individual cultivars may vary across locations due to environmental interactions low broad-sense heritabilities (**Tables 1**, **2**). Cultivars with indicators of slow decomposition in one environment may have indicators of fast decomposition under different environmental conditions. To explore this idea, GGE biplots were created for all traits. For NDF, ADF, cellulose, and hemicellulose, cultivar ranking were generally consistent between the two Pullman environments but showed limited consistency between Central Ferry 2017 and Mansfield 2017, indicating greater variability across the dryland-to-irrigated environmental gradient than between years at the same site. ADL, and carbon exhibited distinct patterns of inter-environment consistency that differed from the Pullman verses other site groupings observed for the fiber constituents.

For a few select cultivars, a correlation was observed between the two Pullman environments when looking at NDF, ADF, cellulose, and hemicellulose but there was no correlation between Central Ferry 2017 and Mansfield 2017 for these traits. Different trends in environmental correlations were observed for ADL, C, and N.

### Marker-trait associations

**Figure 1:**
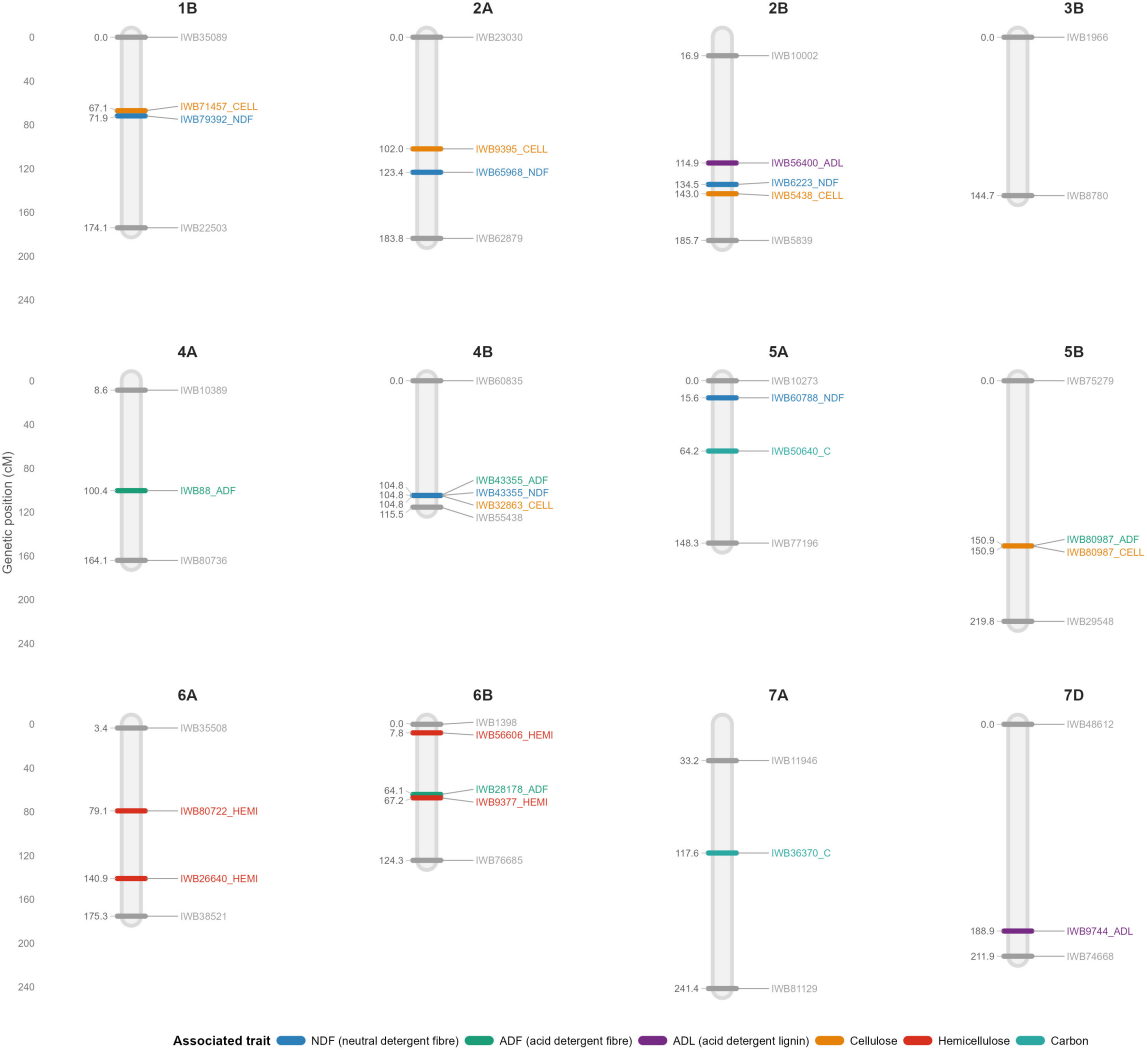
Chromosome map illustrating all 23 MTAs across 12 chromosomes. All MTAs include position (cM) on the left side of each chromosome. The SNP ID and trait are separated by an underscore on the right side of each chromosome. Each trait is designated a different color.

A total of 23 MTAs were detected across 12 wheat chromosomes (**Table 3**; **Figure 1**). Of the 12 chromosomes on which significant MTAs were found, eight had multiple MTAs. Genome B had 14 MTAs detected, whereas eight MTAs were detected on genome A and one MTA was detected on genome D. Chromosomes 2B, 4B, and 6B each had the highest number of detected MTAs (three) whereas chromosomes 3B, 4A, 7A, and 7D each had only one detected MTA. Two MTAs were identified on chromosomes 1B, 2A, 5A, 5B, and 6A. Each trait had significantly associated SNPs and the traits with the highest counts were cellulose and NDF (five each). ADF and hemicellulose followed with four MTAs each, whereas ADL and C each had two. The phenotypic variation explained by the markers ranged from 0.01 to 10.54%.

**Table 3:** Twenty-three SNPs across 12 chromosomes associated with fiber and chemical constituents of straw decomposition.

Trait†	SNP‡	chrom§	pos (cM)¶	Alleles#	p-value	maf††	effect‡‡	PVE§§
CELL	IWB71457	1B	67.14	C/T	4.95E-08	0.22	+	10.21
NDF	IWB79392	1B	71.85	A/G	2.57E-07	0.23	–	4.81
CELL	IWB9395	2A	101.97	T/G	2.04E-07	0.14	+	0.50
NDF	IWB65968	2A	123.35	T/G	9.41E-08	0.46	–	4.13
ADL	IWB56400	2B	114.91	G/A	2.50E-06	0.24	–	0.06
NDF	IWB6223	2B	134.46	T/C	2.84E-06	0.23	+	0.98
CELL	IWB5438	2B	142.99	C/T	1.70E-06	0.45	+	0.02
ADF	IWB88	4A	100.38	A/G	6.54E-07	0.45	+	0.00.01
ADF	IWB43355	4B	104.79	G/A	1.48E-08	0.37	–	0.10.54
CELL	IWB32863	4B	104.79	A/G	7.06E-08	0.35	+	0.07.20
NDF	IWB43355	4B	104.79	G/A	3.86E-10	0.37	–	0.04.07
NDF	IWB60788	5A	15.58	C/A	2.51E-06	0.15	+	0.03.60
C	IWB50640	5A	64.2104	T/C	1.24E-07	0.08	+	0.01.17
ADF	IWB80987	5B	150.93	G/A	3.95E-10	0.31	+	0.04.74
CELL	IWB80987	5B	150.93	G/A	1.64E-09	0.31	+	0.06.46
HEMI	IWB80722	6A	79.08	A/C	9.41E-08	0.35	+	0.03.22
HEMI	IWB26640	6A	140.87	A/G	5.49E-07	0.42	+	0.01.04
HEMI	IWB56606	6B	7.79	T/C	6.29E-09	0.21	–	0.09.46
ADF	IWB28178	6B	64.08	T/C	2.60E-06	0.08	+	0.02.89
HEMI	IWB9377	6B	67.24	C/T	2.48E-06	0.42	+	0.01.01
**C**	IWB36370	7A	117.6079	C/G	5.14E-07	0.17	–	0.04.79
ADL	IWB9744	7D	188.87	G/A	2.10E-09	0.07	–	0.06.19

† NDF, neutral detergent fiber; ADF, acid detergent fiber; ADL, acid detergent lignin; CELL, cellulose; HEMI, hemicellulose; C, carbon.

‡,§,¶ SNP ID, chromosome, and SNP position based on 90K consensus map (Wang et al., 2014).

# Alleles of the marker with minor allele underlined.

†† Minor allele frequency (MAF) in the population.

‡‡ Direction of allelic effect. Selection for the major (underlined) allele will either increase (+) or decrease (-) trait values.

§§ Phenotypic variation explained (PVE) in percentage by the SNP.

The SNP that explained the most phenotypic variation, *IWB43355* (10.5%) and was associated with ADF (**Table 3**). *IWB43355* was also significantly associated with NDF and explained 4.1% of the phenotypic variation. ADF was significantly associated with three other SNPs and one of them, *IWB80987*, was associated with cellulose as well. Cellulose was the trait with the highest number of MTAs (five), and all were identified on different chromosomes (1B, 2A, 2B, 4B, and 5B). The SNP that explained the most phenotypic variation of cellulose was *IWB71457* (10.2%). SNPs *IWB32863* and *IWB80987* explained 7.2% and 6.5% of the phenotypic variation in cellulose, respectively, whereas the other two SNPs, *IWB9395* and *IWB5438*, each explained less than 1%. ADL was associated with marker *IWB9744* that is located on chromosome 7D. This marker explained 6.2% of the phenotypic variation. An eyespot footrot resistance gene, *Pch1*, is also located on chromosome 7D but was not statistically associated with any traits. Marker *IWB56606* was identified on chromosome 6B and was significantly associated with hemicellulose. This SNP explained 9.5% of the phenotypic variation. Three other MTAs were identified with hemicellulose but only explained a small amount of phenotypic variation (1.0-3.2%). Two MTAs were identified with C and explained 1.2 and 4.8% of the phenotypic variation.

Five chromosomal regions of interest were identified. Cellulose was associated with four of these regions whereas ADF and NDF were both associated with three. Markers *IWB71457* (cellulose) and *IWB79392* (NDF) were located closely together on chromosome 1B (positions 67.14 and 71.85, respectively), and markers *IWB6223* (NDF) and *IWB5438* (cellulose) were located less than 9 cM apart on chromosome 2B. On chromosome 4B, markers *IWB32863* and *IWB43355* were both at chromosome position 104.78 and the latter was associated with both NDF and ADF whereas the first had association with cellulose. A semi-dwarfing gene, *Rht-B1*, is also located on chromosome 4B but was not statistically associated with any trait. Marker *IWB80987*, located on chromosome 5B, was associated with both cellulose and ADF. On chromosome 6B, markers *IWB28178* (ADF) and *IWB9377* (hemicellulose) were located very closely, at positions 64.08 and 67.24 respectively, indicating another chromosomal region of interest.

## Discussion

The primary objective for this study was to identify marker-trait associations for the composition of winter wheat straw. We discovered significant MTAs across all traits but the effects of the minor alleles of these SNPs were small. However, considering the very low heritability of these traits, the phenotypic variation explained by several of these markers was fairly high. Nevertheless, the MTAs were widely distributed across the wheat chromosomes and none of the identified SNPs will be useful as standalone markers for marker-assisted selection. They did help to identify several chromosomal regions of interest which may factor into the initial understanding of the genetic composition of these complex traits.

This study found that cellulose, NDF, and ADF were the most useful traits for providing initial insights into the genetic background of straw decomposition. The SNP that explained more phenotypic variation than any other marker, *IWB43355* (10.5%), was associated with ADF and is located on chromosome 4B. This same SNP was also significantly associated with NDF. A second SNP, *IWB32863*, was associated with cellulose and located at the same position on chromosome 4B. There were also strong correlations between all three of these traits (**Supplementary Table 1**). On chromosomes 1B and 2B, two MTAs were detected that both involved cellulose and NDF. These two traits have a strong correlation (0.76-0.93), likely because cellulose is a component of NDF. Similarly, cellulose is a major component of ADF and a strong correlation exists between the two traits (0.87-0.97). This increases our confidence that marker *IWB80987*, which is associated with both ADF and cellulose on chromosome 5B, is marking another region of interest. One other marker associated with ADF is located only 3.2cM away from a marker significantly associated with hemicellulose on chromosome 6B. Due to the proximity of these markers on the genetic map (3.2cM apart), this region may have some influence on fiber production within wheat straw. These five chromosomal loci suggest the possibility of genetic regulation of fiber production in these regions, but this cannot be confirmed at this time.

A strong relationship between straw decomposition and straw strength has been observed (Armbrust, 1980). Significant lodging, or the breaking of straw at the culm or root, is common in wheat and the risk associated fluctuates as the physical strength of the wheat straw changes (Peng et al., 2014). Lignin is known to contribute mechanical strength to plants (Ma, 2009) and has been found in higher quantities in grain cultivars that show high resistance to stem lodging than in cultivars that lodge easily (Wang et al., 2014). Cellulose also contributes significantly to the structural integrity of plants (Liu et al., 2016; Wagner and Wolf, 1998).

Although no MTAs or QTL have previously been identified with the fiber and chemical constituents of winter wheat, a report by Verma et al. (2005) identified QTL for lodging resistance in wheat using single sequence repeat primer pairs as markers. Consistent QTL were detected on chromosomes 4B (Xgwm538–Xgwm6), 4D (Xgwm129–Xgwm608), 6D (Xgwm416–Xgwm14), and 7D (Xgwm44.1–Xgdm150)in both years, while additional QTL detected only in 2001 were located on chromosome 1D (Xgwm216.2–Xgwm458) and 2B (Xgwm374–Xwmc344) (Verma et al., 2005). Following alignment of both marker sets to the IWGSC RefSeq v2.1 wheat reference genome, our SNP markers on chromosomes 2B, 4B, and 7D did not co-localize with the lodging QTL identified by Verma et al. (2005). On chromosome 2B, the Verma 2001 lodging QTL mapped to approximately 173.5 Mb (flanked by Xgwm374 and Xwmc344, which overlap at this position), whereas our 2B SNPs mapped to 716.9 Mb (IWB56400) and 784.5 Mb (IWB5438), clearly distinct regions of the chromosome. On chromosomes 4B and 7D, the SSR-based lodging QTL intervals could not be precisely anchored to the reference genome due to incomplete alignment of one flanking marker (4B) and the very large physical interval between flanking markers (7D), precluding direct positional comparison. Notwithstanding these limitations, no clear co-localization between the lodging QTL of Verma et al. (2005) and the fiber-trait SNPs identified in the present study was observed.

Like lodging resistance, disease resistance has often been linked to increased lignin content in plant cells. An increase in stem lignin content of peppers was observed after *Verticillium dahliae* inoculation (Pomar et al., 2004) and tomato varieties with resistance to *Ralstonia solanacearum* had significantly higher lignin content than susceptible varieties (Mandal et al., 2011). Resistance to eyespot foot rot (*Oculimacula vallundae* and *O. acuformis*) in several dryland cereal cultivars was previously identified to be correlated with high NDF, ADF, and ADL (Stubbs et al., 2009). The correlation between high lignin content and disease resistance is possibly due to the increased development of secondary cell walls which are primarily composed of cellulose, hemicelluloses, and lignin and act as a strong cellular support (Sarkar et al., 2009; Wightman and Turner, 2008; Raven et al., 2005). It is likely that strong secondary cell walls act as a physical barrier to either slow cellular penetration of the pathogen or impede it entirely (Miedes et al., 2014). The eyespot foot rot resistance gene, *Pch1*, located on chromosome 7D (Worland et al., 1988) was included in the genotypic dataset. While the diagnostic SNP marker associated with *Pch1* was not statistically associated with any of our traits, marker *IWB9744*, which was associated with ADL, was identified on chromosome 7D. A relationship between this marker and the *Pch1* gene is unlikely, because we observed recombination between *IWB9744* and the *Pch1* KASP marker.

Environmental influences on fiber and nutrient content in wheat straw can be significant (Grando et al., 2005; Stubbs et al., 2009) as demonstrated by the differences in our phenotypic data across environments (**Table 2**; **Supplementary Figure 1**). Winter wheat cultivars grown in Eastern Washington’s higher rainfall regions have, on average, higher cellulose, NDF, ADF, and ADL than the cultivars grown in the lower rainfall regions. Both years of Pullman (high precipitation) and Central Ferry (irrigated) had higher NDF, ADF, ADL, and cellulose than Mansfield (low precipitation), reflecting the concept that lower precipitation generally results in straw with low NDF and ADF, both indicators of rapid decomposition (Stubbs et al., 2009). Environmental factors other than precipitation are also known to affect lignification such as air temperature (Olenichenko and Zagoskina, 2005) and N deficiencies (Fritz et al., 2006). Soil salinity (Al-Hakimi and Hamada, 2001) can also impact lignin, cellulose, and hemicellulose production in wheat straw.

Although the lower precipitation site generally resulted in lower fiber values than the high precipitation and irrigated sites, the same trend was not observed for hemicellulose. High hemicellulose content has been linked to rapid decomposition (Wagner and Wolf, 1998) and is possibly a result of lower precipitation and increased drought stress. However, the method that was used to determine NDF, ADF, and ADL (from which cellulose and hemicellulose are derived) of a single cultivar is based upon percentages relative to the other constituents within the same sample. Therefore, it is possible that a higher hemicellulose value comes as a result of developmental hindrance of lignified secondary cell walls due to stress. In short, high hemicellulose percentage may simply be a reflection of low lignin content.

There have been, however, mixed results found in literature concerning lignin synthesis during drought stress. Lignin content was increased in switchgrass in one experiment and decreased in a second experiment by the same authors while under drought stress (Jiang et al., 2012), whereas lignin production was induced in perennial ryegrass when water was withheld (Lee et al., 2012). Our results clearly indicate a decrease in ADL when water stress becomes a factor.

## Conclusion

To facilitate the adoption of no-till systems across wheat producing regions, the decomposition characteristics of wheat straw from numerous cultivars need to be well understood. Decomposition potential of individual cultivars appears to be dependent on the environment, possibly due to the varying production inputs like fertilizer. The genetic composition of wheat straw decomposition constituents is complex, yet the phenotypic variation explained by most of the significant markers was actually quite high. The results help to estimate the usefulness of each trait for similar future studies. Carbon was the least useful for identifying regions of interest as only three SNPs explaining very little of the phenotypic variation were associated with the two traits. Cellulose, ADF, and NDF contributed the most to identifying chromosomal regions of interest and provided valuable information regarding the genetic composition of fiber production in straw. This study continues to reveal genetic information about the architecture of constituents associated with straw decomposition that will be important to help convert more growing regions to no-till production farming.

## Data Availability

The datasets presented in this study can be found in online repositories. The names of the repository/repositories and accession number(s) can be found in the article/**Supplementary Material**.

## References

[B1] Aher G. Cihacek L. J. Cooper K. (2017). An evaluation of C and N on fresh and aged crop residue from mixed long-term no-till cropping systems. J. Plant Nutr. 40, 177–186. doi: 10.1080/01904167.2016.1201505 37339054

[B2] Al-Hakimi A. Hamada A. M. (2001). Counteraction of salinity stress on wheat plants by grain soaking in ascorbic acid, thiamin or sodium salicylate. Biol. Plantarium 44, 253–261. doi: 10.1023/a:1010255526903 41886696

[B3] Armbrust D. V. (1980). Tests to determine wheat straw decomposition. Agron. J. 72, 399–401. doi: 10.2134/agronj1980.00021962007200020036x

[B4] (2018). Wheat & Small Grains. Available online at: http://smallgrains.wsu.edu (Accessed July 1, 2026).

[B5] Bates D. Mächler M. Bolker B. Walker S. (2015). Fitting linear mixed-effects models using lme4. J. Stat. Software 67, 1–48. doi: 10.18637/jss.v067.i01

[B6] Bernal E. (2023). GGEBiplotGUI: Interactive visualization system for GGE biplots ( R package version 1.0-9). Available online at: https://cran.r-project.org/src/contrib/Archive/GGEBiplotGUI/.

[B7] Douglas C. L. Allmaras R. R. Rasmussen P. E. Ramig R. E. Roager N. J. (1980). Wheat straw composition and placement effects on decomposition in dryland agriculture of the Pacific Northwest. Soil Sci. Soc Am. J. 44, 833–837. doi: 10.2136/sssaj1980.03615995004400040035x

[B8] Esposito S. Taranto F. Vitale P. Ficco D. B. M. Colecchia S. A. Stevanato P. . (2022). Unlocking the molecular basis of wheat straw composition and morphological traits through multi-locus GWAS. BMC Plant Biol. 22, 519. doi: 10.1186/s12870-022-03900-6 36344939 PMC9641881

[B9] Feekes W. (1941). “ De Tarwe en haar milieu,” in Tech. Tarwe Comm, (Groningen: Technische Tarwe Commissie) vol. XVII. , 560–561.

[B10] Frazier B. E. McCool D. K. Engle C. F. (1983). Soil erosion in the Palouse: An aerial perspective. J. Soil Water Conserv. 38, 70–74. doi: 10.1080/00224561.1983.12436251 37339054

[B11] Fritz C. Palacios-Rojas N. Feil R. Stitt M. (2006). Regulation of secondary metabolism by the carbon-nitrogen status in tobacco: nitrate inhibits large sectors of phenylpropanoid metabolism. Plant J. 46, 533–548. doi: 10.1111/j.1365-313x.2006.02715.x 16640592

[B12] Froese P. S. Carter A. H. (2016). Single nucleotide polymorphisms in the wheat genome associated with tolerance of acidic soils and aluminum toxicity. Crop Sci. 56, 1662–1677. doi: 10.2135/cropsci2015.10.0629

[B13] Gazulla M. F. Rodrigo M. Orduña M. Gómez C. M. (2012). Determination of carbon, hydrogen, nitrogen, and sulfur in geological materials using elemental analysers. Geostand. Geoanal. Res. 36, 1397–1402. doi: 10.1111/j.1751-908x.2011.00140.x 40046247

[B14] Grando S. Baum M. Ceccarelli S. Goodchild A. Jaby El-Haramein F. Jahoor A. . (2005). QTLs for straw quality characteristics identified in recombinant inbred lines of Hordeum vulgare x H. spontaneum cross in a Mediterranean environment. Theor. Appl. Genet. 110, 688–695. doi: 10.1007/s00122-004-1894-3 15678328

[B15] Holland J. B. Nyquist W. E. Cervantes-Martínez C. T. (2003). “ Estimating and interpreting heritability for plant breeding: An update,” in Plant Breeding Reviews, Volume 22. Ed. Jules J. ( John Wiley & Sons, Inc, Hoboken), 9–112.

[B16] Hothorn T. Bretz F. Westfall P. (2008). Simultaneous inference in general parametric models. Biom. J. 50, 346–363. doi: 10.1002/bimj.200810425 18481363

[B17] Jernigan K. L. Godoy J. V. Huang M. Zhou Y. Morris C. F. Garland-Campbell K. A. . (2018). Genetic dissection of end-use quality traits in adapted soft white winter wheat. Front. Plant Sci. 9. doi: 10.3389/fpls.2018.00271 29593752 PMC5861628

[B18] Jiang Y. Yao Y. Wang Y. (2012). Physiological response, cell wall components, and gene expression of switchgrass under short-term drought stress and recovery. Crop Sci. 52, 2718–2727. doi: 10.2135/cropsci2012.03.0198

[B19] Joshi A. K. Kumar U. Mishra V. K. Chand R. Chatrath R. Naik R. . (2019). Variations in straw fodder quality and grain–straw relationships in a mapping population of 287 diverse spring wheat lines. Field Crops Res. 243, 107627. doi: 10.1016/j.fcr.2019.107627 31853164 PMC6894307

[B20] Kravchenko A. G. Thelen K. D. (2007). Effect of winter wheat crop residue on no-till corn growth and development. Agron. J. 99, 549–555. doi: 10.2134/agronj2006.0192

[B21] Large E. C. (1954). Growth stages in cereals illustrations of the Feekes scale. Plant Pathol. 3, 128–129. doi: 10.1111/j.1365-3059.1954.tb00716.x 40046247

[B22] Lee B. Muneer S. Jung W. Avice J. Ourry A. Kim T. (2012). Mycorrhizal colonization alleviates drought-induced oxidative damage and lignification in the leaves of drought-stressed perennial ryegrass (Lolium perenne). Physiol. Plantarum. 145, 440–449. doi: 10.1111/j.1399-3054.2012.01586.x 22289111

[B23] Liu X. Huang M. Fan B. Buckler E. S. Zhang Z. (2016). Iterative usage of fixed and random effect models for powerful and efficient genome-wide association studies. PloS Genet. 12, e1005767. doi: 10.1371/journal.pgen.1005767 26828793 PMC4734661

[B24] Liu W. Naruoka Y. Miller K. Garland-Campbell K. A. Carter A. H. (2018). Characterizing and validating stripe ruse resistance loci in US Pacific Northwest winter wheat accessions (Triticum aestivum L.) by genome-wide association and linkage mapping. Plant Genome 11, 1–16. doi: 10.3835/plantgenome2017.10.0087 29505636 PMC12809922

[B25] Ma Q. (2009). The expression of caffeic acid 3-O-methyltransferase in two wheat genotypes differing in lodging resistance. J. Exp. Bot. 61, 2763–2771. doi: 10.1093/jxb/erp132 19451187 PMC2692018

[B26] Maaz T. M. Schillinger W. F. MaChado S. Brooks E. Johnson-Maynard J. L. Young L. E. . (2017). Impact of climate change adaptation strategies on winter wheat and cropping system performance across precipitation gradients in the Inland Pacific Northwest, USA. Front. Environ. Sci. 5, 23. doi: 10.3389/fenvs.2017.00023

[B27] Mandal S. Das R. K. Mishra S. (2011). Differential occurrence of oxidative burst and antioxidative mechanism in compatible and incompatible interactions of Solanum lycopersicum and Ralstonia solanacearum. Plant Physiol. Bioch. 49, 117–123. doi: 10.1016/j.plaphy.2010.10.006 21093281

[B28] McCann M. Rose J. (2010). Blueprints for building plant cell walls. Plant Physiol. 153, 365. doi: 10.1104/pp.110.900324 20522725 PMC2879777

[B29] Miedes E. Vanholme R. Boerjan W. Molina A. (2014). The role of the secondary cell wall in plant resistance to pathogens. Front. Plant Sci. 5. doi: 10.3389/fpls.2014.00358 25161657 PMC4122179

[B30] Nurbekov A. Kosimov M. Islamov S. Khaitov B. Qodirova D. Yuldasheva Z. . (2024). No-till, crop residue management and winter wheat-based crop rotation strategies under rainfed environment. Front. Agron. 6. doi: 10.3389/fagro.2024.1453976

[B31] Olenichenko N. A. Zagoskina N. V. (2005). Response of winter wheat to cold: Production of phenolic compounds and L-phenylalanine ammonia lyase activity. Appl. Biochem. Microbiol. 41, 681–685. doi: 10.1007/s10438-005-0109-2 16358760

[B32] Papendick R. I. Lindstrom M. J. Cochran V. L. (1973). Soil mulch effects on seedbed temperature and water during fallow in Eastern Washington. Soil Sci. Soc Am. Proc. 37, 307–314. doi: 10.2136/sssaj1973.03615995003700020039x

[B33] Pearce S. Saville R. Vaughan S. P. Chandler P. M. Wilhelm E. P. Sparks C. A. . (2011). Molecular characterization of Rht-1 dwarfing genes in hexaploid wheat. Plant Physiol. 157, 1820–1831. doi: 10.1104/pp.111.183657 22013218 PMC3327217

[B34] Peng D. Chen X. Yin Y. Lu K. Yang W. Tang Y. . (2014). Lodging resistance of winter wheat (Triticum aestivum L.): Lignin accumulation and its related enzymes activities due to the application of paclobutrazol or gibberellin acid. Field Crop Res. 157, 1–7. doi: 10.1016/j.fcr.2013.11.015 38826717

[B35] Pomar F. Novo M. Bernal M. A. Merino F. Barceló A. R. (2004). Changes in stem lignins (monomer composition and crosslinking) and peroxidase are related with the maintenance of leaf photosynthetic integrity during Verticillium wilt in Capsicum annuum. New Phytol. 163, 111–123. doi: 10.1111/j.1469-8137.2004.01092.x 33873795

[B36] Raven P. H. Evert R. F. Eichhorn S. E. (2005). “ Biology of plants,” in Biology of Plants, 7th Ed, vol. 7. ( W. H. Freeman and Company, New York).

[B37] Rosario-Lebron A. Leslie A. W. Yurchak V. L. Chen G. Hooks C. R. R. (2019). Can winter cover crop termination practices impact weed suppression, soil moisture, and yield in no-till soybean [Glycine max (L.) Merr.]? Crop Prot. 116, 132–141. doi: 10.1016/j.cropro.2018.10.020 38826717

[B38] Ruan L. Robertson G. P. (2020). No-till establishment improves the climate benefit of bioenergy crops on marginal grasslands. Soil Sci. Soc Am. J. 84, 1280–1295. doi: 10.1002/saj2.20082 41531421

[B39] Sarkar P. Bosneaga E. Auer M. (2009). Plant cell walls throughout evolution: towards a molecular understanding of their design principles. J. Exp. Bot. 60, 3615–3635. doi: 10.1093/jxb/erp245 19687127

[B40] Schillinger W. F. Papendick R. I. (2008). Then and now: 125 years of dryland wheat farming in the Inland Pacific Northwest. Agron. J. 100, S166–S182. doi: 10.2134/agronj2007.0027c

[B41] Sharratt B. Feng G. Wendling L. (2007). Loss of soil and PM10 from agricultural fields associated with high winds on the Columbia Plateau. Earth Surf. Proc. Land. 32, 621–630. doi: 10.1002/esp.1425 41531421

[B42] Stubbs T. L. Kennedy A. C. Reisenauer P. E. Burns J. W. (2009). Chemical composition of residue from cereal crops and cultivars in dryland ecosystems. Agron. J. 101, 538–545. doi: 10.2134/agronj2008.0107x

[B43] Tang Y. Liu X. Wang J. Li M. Wang Q. . (2016). GAPIT version 2: an enhanced integrated tool for genomic association and prediction. Available online at: https://www.nrcs.usda.gov/Internet/FSE_DOCUMENTS/nrcseprd331820.pdf (Accessed 15 Nov. 2017). 10.3835/plantgenome2015.11.0120 27898829

[B44] USDA-NRCS (2017). Web Soil Survey. Available online at: https://websoilsurvey.sc.egov.usda.gov/App/HomePage.htm (Accessed July 1, 2026).

[B45] van Soest P. J. Robertson J. B. Lewis B. A. (1991). Methods for dietary fiber, neutral detergent fiber, and nonstarch polysaccharides in relation to animal nutrition. J. Dairy Sci. 74, 3583–3597. doi: 10.3168/jds.s0022-0302(91)78551-2 1660498

[B46] Verma V. Worland A. J. Savers E. J. Fish L. Caligari P. D. S. Snape J. W. . (2005). Identification and characterization of quantitative trait loci related to lodging resistance and associated traits in bread wheat. Plant Breed. 124, 234–241. doi: 10.1111/j.1439-0523.2005.01070.x 40046247

[B47] Wagner G. H. Wolf D. C. (1998). “ Carbon transformations and soil organic matter formation,” in Principles and Applications of Soil Microbiology. Eds. Sylvia D. M. Fuhrmann J. J. Hartel P. G. Zuberer D. A. ( Prentice Hall, Upper Saddle River, NJ), 218–258.

[B48] Wang C. Ruan R. Yuan X. Hu D. Yang H. Li Y. . (2014). Relationship between lignin metabolism and lodging resistance of culm in buckwheat. J. Agr. Sci. 9, 29–36. doi: 10.5539/jas.v6n9p29

[B49] Wang S. Wong D. Forrest K. Allen A. Chao S. Huang B. E. . (2014). Characterization of polyploid wheat genomic diversity using a high-density 90,000 sing nucleotide polymorphism array. Plant Biotechnol. J. 12, 787–796. doi: 10.1111/pbi.12183 24646323 PMC4265271

[B50] Wightman R. Turner S. R. (2008). The roles of the cytoskeleton during cellulose deposition at the secondary cell wall. Plant J. 54, 794–805. doi: 10.1111/j.1365-313x.2008.03444.x 18266917

[B51] Williams J. D. Wuest S. B. Long D. S. (2014). Soil and water conservation in the Pacific Northwest through no-tillage and intensified crop rotations. J. Soil Water Conserv. 69, 495–504. doi: 10.2489/jswc.69.6.495

[B52] Worland A. J. Law C. N. Hollins T. W. Koebner R. M. D. Giura A. (1988). Location of a gene for resistance to eyespot (Pseudocercosporella herpotrichoides) on chromosome 7D of bread wheat. Plant Breed. 101, 43–51. doi: 10.1111/j.1439-0523.1988.tb00265.x 40046247

