# Supplementary material for: Wheat straw decomposition constituents: a genome-wide association study and environmental influence analysis
Source: Front Plant Sci. 2026 Jul 16;17:1837289. doi: 10.3389/fpls.2026.1837289 (PMC13421105; doi:10.3389/fpls.2026.1837289)

Table S1 Correlations for NDF, ADF, ADL, Cellulose (CELL), Hemicellulose (HEMI), carbon (C), and nitrogen (N) across four environments; Pullman 2016, Pullman 2017, Central Ferry 2017, and Mansfield 2017.

Pullman 2016						
	NDF	ADF	ADL	CELL	HEMI	C
NDF	1	0.8576	0.4506	0.8653	0.3349	0.3916
ADF		1	0.6747	0.94	-0.1973	0.3149
ADL			1	0.3824	-0.3772	0.2263
CELL				1	-0.0728	0.2895
HEMI					1	0.1695
C						1
Pullman 2017						
	NDF	ADF	ADL	CELL	HEMI	C
NDF	1	0.7279	0.2501	0.7611	0.2809	0.2262
ADF		1	0.6227	0.869	-0.4536	0.1886
ADL			1	0.1541	-0.5467	0.0901
CELL				1	-0.2271	0.1813
HEMI					1	0.03
C						1
Central Ferry 2017						
	NDF	ADF	ADL	CELL	HEMI	C
NDF	1	0.9086	0.5668	0.9289	0.2276	0.1265
ADF		1	0.7621	0.9732	-0.1999	0.0935
ADL			1	0.5928	-0.4471	0.0584
CELL				1	-0.0898	0.0955
HEMI					1	0.0789
C						1
Mansfield 2017						
	NDF	ADF	ADL	CELL	HEMI	C
NDF	1	0.8787	0.4499	0.8904	0.6954	0.1922
ADF		1	0.652	0.9649	0.2679	0.1815
ADL			1	0.4301	-0.0737	0.0615
CELL				1	0.3445	0.1949
HEMI					1	0.1145
C						1

Figure S1 Boxplots displaying the minimum, first quartile, median, third quartile, and maximum. Traits include (a) NDF, (b) ADF, (c) ADL, (d) Cellulose, (e) Hemicellulose, and (f) C. Environments are Central Ferry 2017 (CF17), Mansfield 2017 (Man17), Pullman 2016 (Pul16), and Pullman 2017 (Pul17).

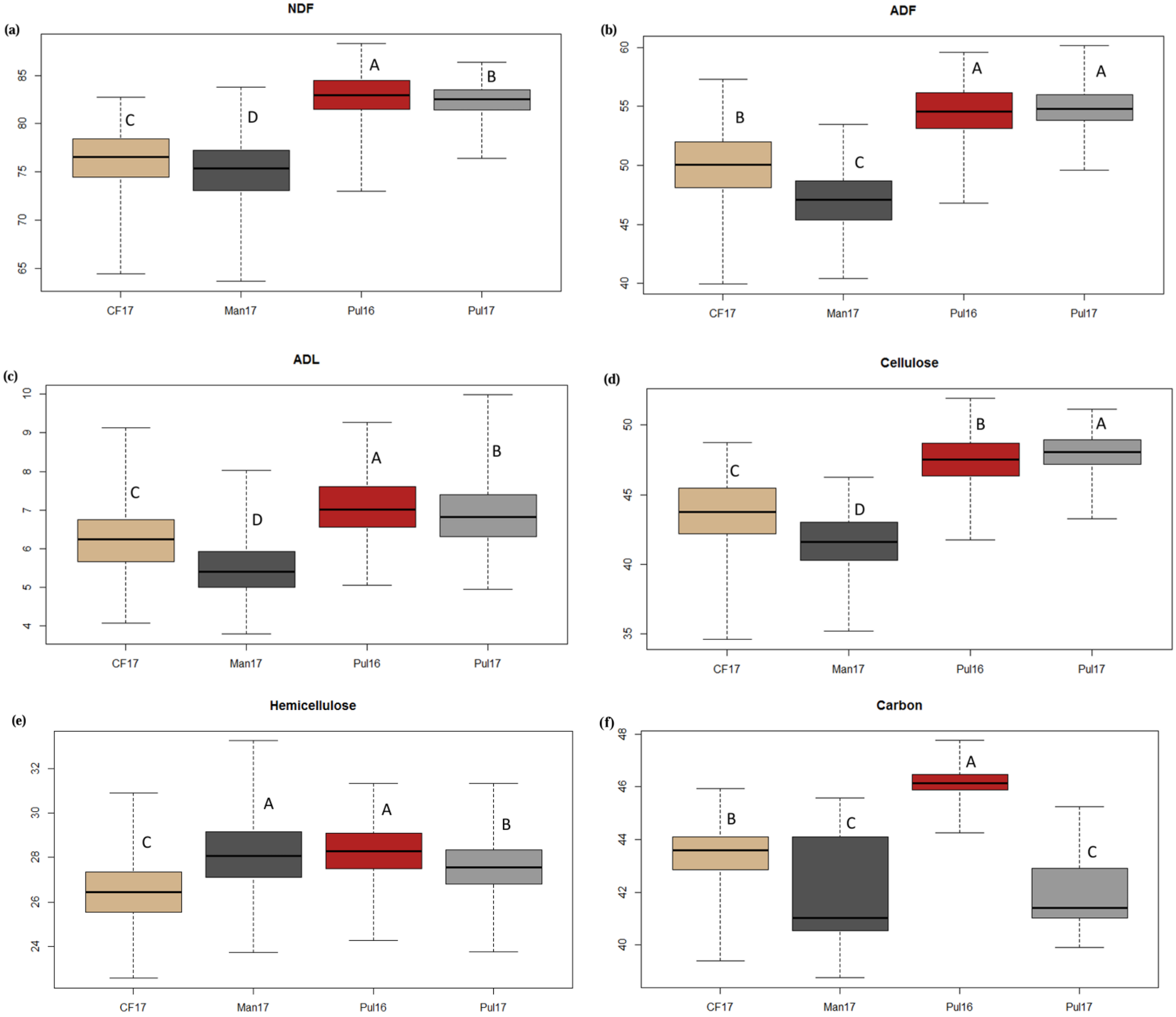

Supplement: Supplementary file 1 [file SupplementaryFile1.pdf]
